# Biomarkers of Individual Health Status Provide a Biological Signature of the Whole Person Living with Chronic Pain

**DOI:** 10.3390/biomedicines14102217

**Published:** 2026-09-30

**Authors:** Kimberly T. Sibille, Angela M. Mickle, Katrina R. Hamilton, Akemi T. Wijayabahu, Steven D. Pratscher, Lydia G. Pezzullo, Karyn A. Esser, Stephen A. Coombes, Jared J. Tanner

**Affiliations:** 1 Department of Physical Medicine & Rehabilitation, College of Medicine, University of Florida, 2004 Mowry Rd, Gainesville, FL 32610, USA; 2 Department of Psychology, Ohio University, 200 Porter Hall, Athens, OH 45701, USA; hamiltonk@ohio.edu; 3 Division of Cancer Epidemiology and Genetics, National Cancer Institute, National Institutes of Health, 9609 Medical Center Drive, Rockville, MD 20850, USA; akemi.wijayabahu@nih.gov; 4 Division of Palliative Medicine, Department of Internal Medicine, College of Medicine, The Ohio State University, 3650 Olentangy River Rd, Columbus, OH 43214, USA; 5 Department of Physiology and Aging, College of Medicine, University of Florida, 1345 Center Drive M544, Gainesville, FL 32611, USA; 6 Department of Applied Physiology and Kinesiology & Department of Biomedical Engineering, College of Health and Human Performance, University of Florida, 1864 Stadium Rd, Gainesville, FL 32611, USA; 7 Department of Clinical and Health Psychology, College of Public Health and Health Professions, University of Florida, 1225 Center Dr, Gainesville, FL 32611, USA

**Keywords:** chronic pain, biomarkers of individual health status, allostatic load, biological signature, whole person health

## Abstract

Enormous investments in time and resources have been committed in the pursuit of reliable pain biomarkers and clinical endpoints to inform interventional investigations and patient care. Although there has been noteworthy progress, the field remains with limited tools to address the pressing needs of the clinical and research communities. Broadening the view and shifting the conceptual approach from a focus on pain mechanisms to include measures of biological individual health status might provide valuable information that has thus far been missed. Chronic pain is experienced within a complex and multifaceted array of life experiences. It is well established that numerous biopsychosocial factors are associated with variability in outcomes among individuals with chronic pain. Biomarkers that can capture the whole person milieu of an individual living with chronic pain would be highly informative. An overview of the theoretically grounded and evidence-based model, allostatic load, is provided. A summary of compelling evidence involving biomarkers of individual health status, measures of allostatic load, in chronic pain research follows. Next, possible complementary roles for biomarkers of individual health status in pain biomarker efforts are presented. Lastly, necessary next steps and future directions are identified. Although investigations are still in the early stages, biomarkers of individual health status offer promising potential to help (1) explain clinical heterogeneity, (2) identify those individuals more vulnerable to worse outcomes, and, with prospective validation, possibly (3) elucidate treatment response variability and (4) serve as clinical monitoring and surrogate endpoints.

## 1. Introduction

A lack of reliable biomarkers and clinical endpoints are major obstacles delaying progress in chronic pain research, clinical trials, and patient care [1,2,3]. Over the past ten years, several steps have been taken to accelerate scientific progress and address persisting deficits. In 2015, the U.S. Food and Drug Administration (FDA) and the National Institutes of Health (NIH) Biomarker Working Group created the Biomarker, EndpointS, and other Tools (BEST) Resource. The purpose of the group was to develop an infrastructure to align understanding and promote consistency in biomarker communications across clinical research investigations [4]. In the BEST glossary, a biomarker is “a defined characteristic that is measured as an indicator of normal biological processes, pathogenic processes, or biological responses to an exposure or intervention, including therapeutic interventions” [4]. The biomarker working group classified eight different types of biomarkers: susceptibility/risk, diagnostic, monitoring, prognostic, predictive, multicomponent, response, and safety [4].

In 2018, the Discovery and Validation of Biomarkers to Develop Non-Addictive Therapeutics for Pain workshop was convened by the National Institutes of Health. The intention of the workshop was to identify next steps in the development of biomarkers and clinical endpoints for pain [5]. More recently, in 2025, the Executive Committee for the HEAL (Helping to End Addiction Long-term) Initiative developed a Pain Strategic Plan. The plan summarizes progress over the previous five years of HEAL funding and identifies strategic targets moving forward [6,7]. Priority E specifically identifies the need to “develop biomarkers for predicting treatment response, safety, target engagement, and/or that may serve as surrogate endpoints in clinical trials [7].”

Advances in pain biomarker research thus far span a breadth of measures across different levels of biological functioning to include cellular (e.g., genetic, epigenetic, metabolomics, proteomics), organ (e.g., brain neuroimaging and electrophysiological measures), systems (e.g., inflammatory, nociceptive, neurochemical), and organism (e.g., physiological and behavioral measures) [8,9,10,11,12,13,14]. Computational strategies involving artificial intelligence and machine learning techniques are also being leveraged to inform the identification of pain biomarker signatures [8,15,16,17]. Importantly, across investigations, the individual heterogeneity entanglement persists [18,19,20,21].

Pain biomarker research has primarily focused on pain mechanisms and biological indicators of specific pain characteristics. Evidence indicates that a broader view, a conceptual shift, and a combined approach may help address some pain biomarker limitations. Even with the eventual identification of susceptibility, diagnostic, predictive, and prognostic pain biomarkers, a full resolution to individual variability in outcomes is not anticipated. As evident in other health conditions, despite well-established biomarkers in cancer and cardiovascular disease, the pursuit for improved biomarkers continues as heterogeneity in clinical outcomes remains [22,23]. Health conditions, including chronic pain, occur within the complex array of an individual’s life experiences. A plethora of research shows that the onset and severity of chronic pain is associated with numerous intrinsic and extrinsic factors [24,25,26]. Biomarkers that convey the “whole person milieu,” i.e., the cumulative biological representation of an individual’s life experiences, might help disentangle between-person differences that persist across studies.

Allostasis is a term used to describe the ongoing process of how an individual’s biological systems adapt to internal and external stimuli [27]. Allostatic load is a theoretically based and empirically informed concept that captures the “dynamic interaction of a person’s biology and biography” [28]. Although often recognized as an indicator of chronic stress, allostatic load is broader in nature and better conceptualized as a balance of risk and protective factors from endogenous and exogenous sources. Allostatic load can be interpreted from measures that convey an individual’s biological health status [29]. Thus, adaptive biological functioning would indicate higher health reserves and low allostatic load, while compromised biological functioning would indicate lower health reserves and high allostatic load/overload. High allostatic load is associated with a delayed response to treatment and worse health outcomes [29,30,31]. Biological measures that convey the “unique health profile of each individual” are identified as biomarkers of individual health status [28,29]. An array of studies convey compelling evidence that biomarkers of individual health status help explain the clinical heterogeneity observed in individuals with chronic pain [32,33,34,35,36,37,38].

We provide an overview of how allostatic load helps frame a “whole person” approach that could complement pain biomarker investigations [39]. Next, we present a summary of findings involving biomarkers of individual health status in chronic pain research. Following the summary, the applicability of the allostatic load model and the utility and possible contributions of biomarkers of individual health status to pain biomarker development will be reviewed. Lastly, we identify the necessary next steps for future investigations involving biomarkers of individual health status and explore considerations in the development of multicomponent pain biomarkers and “composite pain signature” approaches [7,8].

## 2. Relevant Sections

### 2.1. A Broader View

Current pain phenotyping efforts have a limited role in explaining clinical heterogeneity and associate poorly with biological measures [40,41,42]. Life experiences influence health outcomes [25]. It is clearly established that socioenvironmental, behavioral, and psychosocial factors contribute to the inter-individual variability in chronic pain. A consistent body of findings show that socioenvironmental factors such as lower education and income, limited access to healthcare, and greater experiences of discrimination are associated with higher clinical and experimental pain and worse health outcomes in individuals with chronic pain [25,26,43,44,45]. Inclusion of socioenvironmental factors in study designs has helped explain observed differences in pain-related outcomes [34,46,47,48]. Similarly, behavioral and psychosocial factors are associated with clinical pain, physical function, and mortality risk [24,25,49,50]. Protective factors such as regular exercise, a healthy weight, quality sleep, being a non-smoker, and social support, are all associated with better pain-related outcomes [32,37,38].

### 2.2. Conceptual Shift

The relationships between chronic pain and socioenvironmental, psychosocial, and behavioral factors have been assessed in research investigations. However, to truly improve health outcomes, a combined understanding of contributing factors is needed. As noted by Dr. Langevin, former director of the National Center for Complementary and Integrative Health, “we want to stop thinking about biologic, behavioral, social, and environmental factors as separate and instead look at them all together… The point of whole person health is to think about the integration of the whole person” (p. 345) [39].

It is well recognized that humans are born with a foundation of genetically based, intrinsic, predisposing biological factors that underlie the internal functioning of the individual. These biologically based factors are dynamically influenced and altered by life experiences and exposures. The bidirectional interaction between biology and biography begins at conception and continues until death [51,52,53,54,55]. Intrinsic and extrinsic factors communicate through the activation of biological processes [52,53,54,55,56,57,58]. Life experiences are transposed into biological signals stimulating processes that drive changes across different levels of functioning (i.e., molecular, cellular, tissue, organ, organism) which influence health outcomes across the life course [55,56,57,59]. Research has advanced to a stage where numerous factors, across differing biological levels of analysis, have been associated with the onset and severity of chronic pain. Importantly, the relevance of socioenvironmental, behavioral, and psychosocial factors extends beyond chronic pain conditions, contributing to whole person health [59,60,61,62,63,64,65,66,67,68].

**Figure 1:**
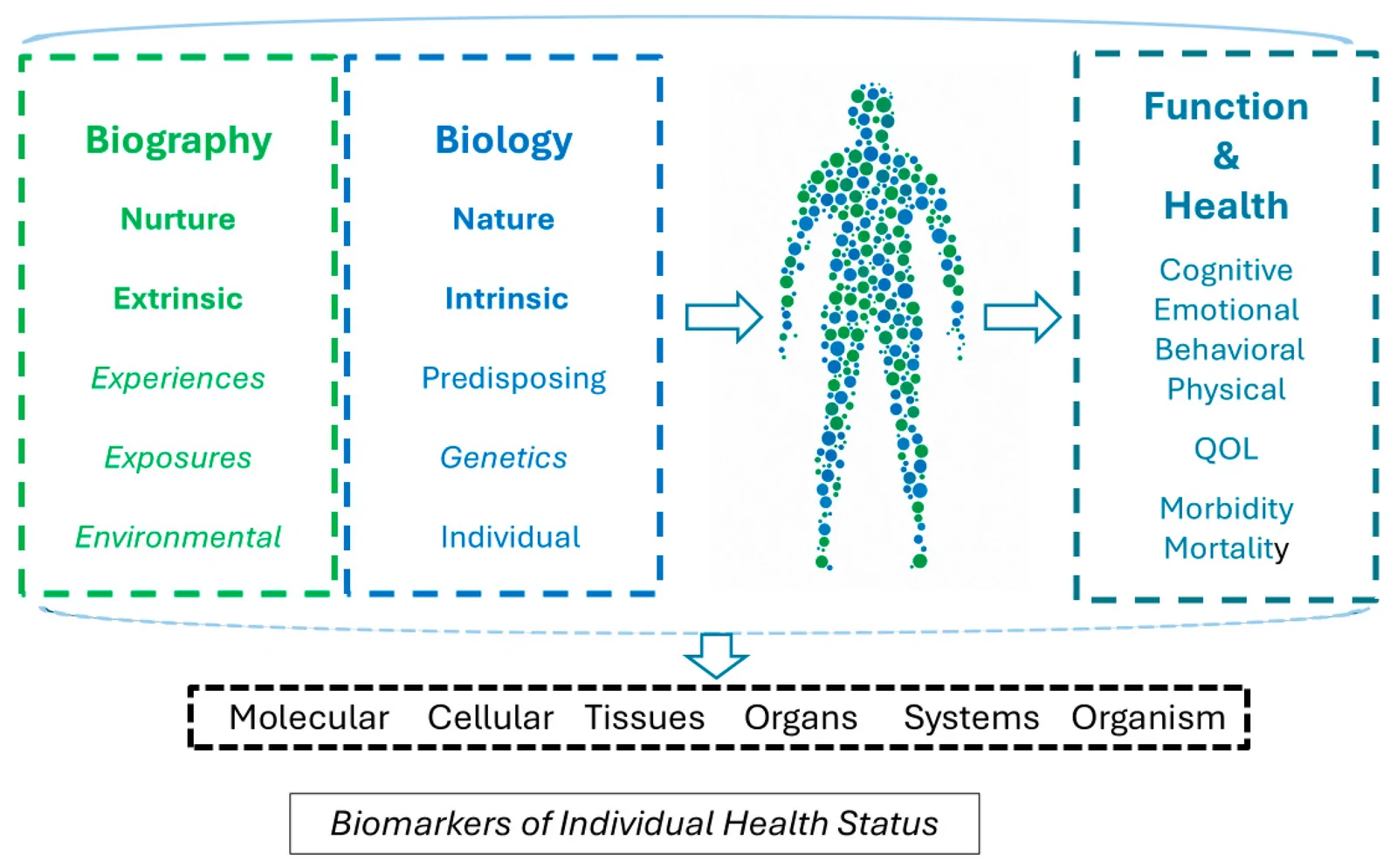
Functional depiction of the relationships between intrinsic and extrinsic factors and their dynamic influence on levels of biological analysis, organism functioning, and health outcomes. Biomarkers of individual health status convey the unique biological signature resulting from the whole person life experience.

Figure 1 depicts the described relationships between intrinsic and extrinsic factors and their predictive relationship with biological and clinical health outcome measures. Consistent with a whole person health approach, potential predictor factors are numerous, including sex, age, comorbidities, medications, health condition severity (chronic pain stage), socioenvironmental factors, and behavioral/psychosocial factors.

**Figure 2:**
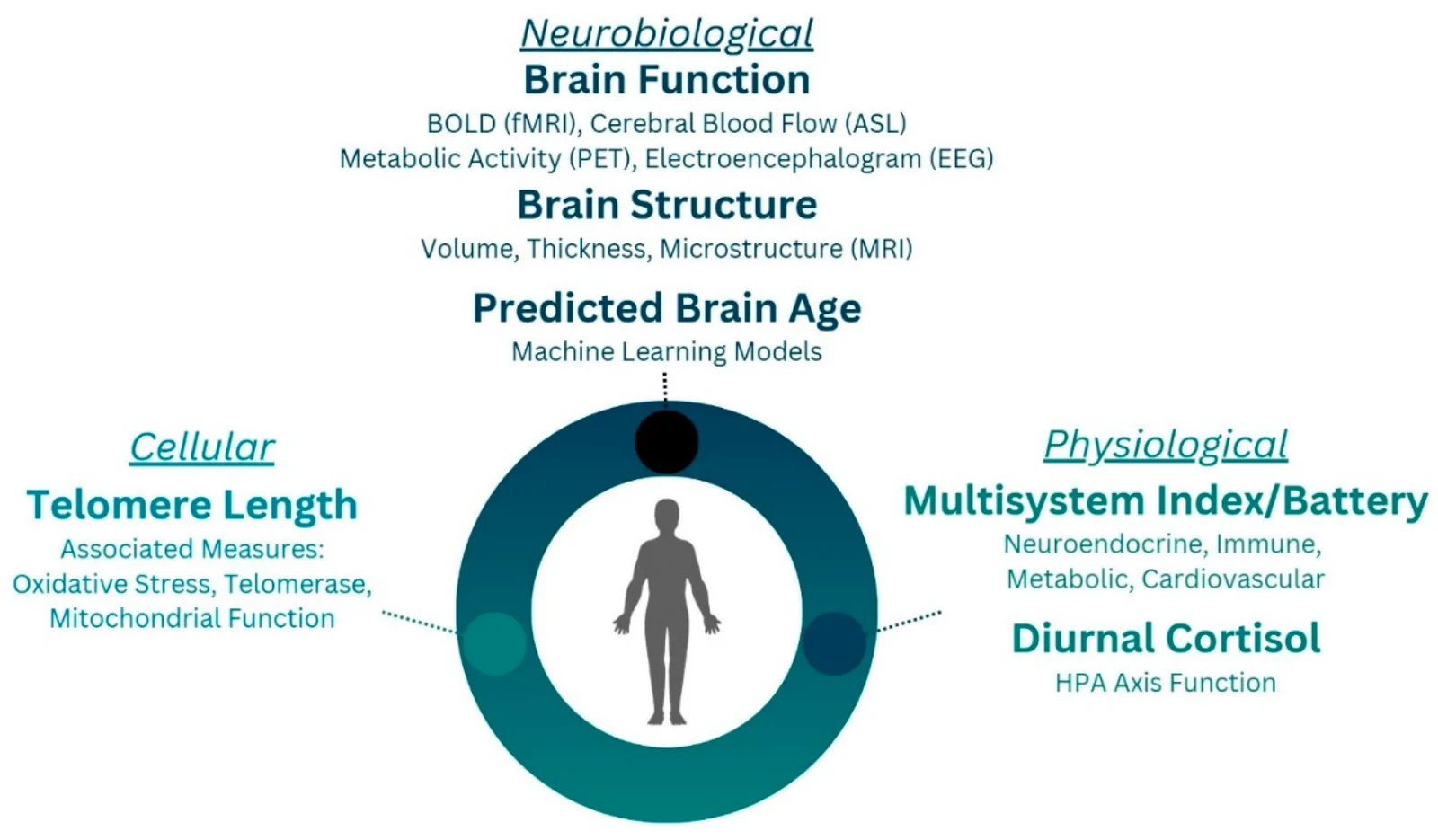
Different biomarkers of individual health status across levels of biological organization.

Recent advances in the understanding of the biological embedding of socioenvironmental, behavioral, and psychosocial factors and their influence on health outcomes have prompted renewed interest in the concept of allostatic load [58,69,70]. The allostatic load theory helps to explain the biological interface resulting from an individual’s predisposing factors, life exposures, and associated responses [28,69,71,72,73,74,75,76]. Collective evidence demonstrates allostatic load has application across all levels of biological analysis from molecular processes to health-related outcomes [29,77,78,79,80,81,82,83,84]. Applying the allostatic load conceptualization to chronic pain research investigations provides a framework to understand relationships between combined biopsychosocial or “whole person” experiences and biological outcome measures in individuals living with chronic pain. Biological measures reflecting allostatic load have been referred to as biomarkers of individual health status and include brain function, brain structure, a clinical multisystem index, diurnal cortisol, and telomere length among others, see Figure 2 [29,85,86,87,88,89,90,91,92,93]. 

**Figure 3:**
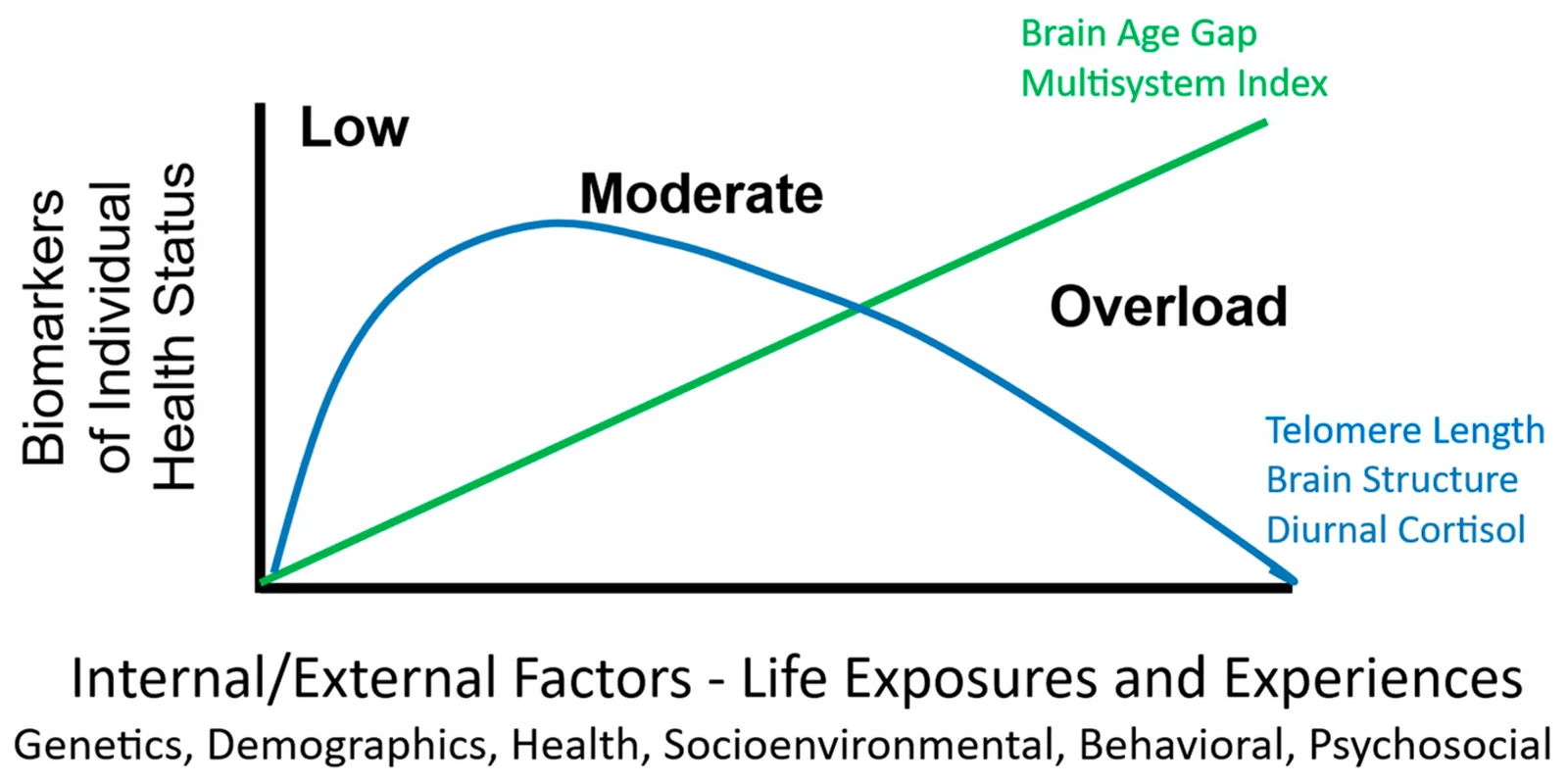
Heuristic model for interpreting levels of allostatic load based on findings across different biomarkers of individual health status (accounting for linear and non-linear distributions).

Depicted in Figure 3, low allostatic load is evidenced by biological measures indicating clinically normal ranges suggesting an adaptive, positive health status. Moderate allostatic load is distinguished by biomarkers showing elevated measures and signs of dysregulation, signifying a compromised health status. High allostatic load, also known as allostatic overload, is demonstrated by biomarkers that are either flattened or blunted, suggestive of accelerated aging (e.g., short telomeres or predicted structural brain age is significantly older than chronological brain age), indicating a depleted health status [31,74,75,76,94]. These distinctions are clinically relevant as biomarkers of individual health status help explain variance in health outcomes including morbidity and mortality and changes in response to therapeutic interventions [29,31,64,80,95,96,97]. Thus, biomarkers reflecting the biological interface of an individual’s life experiences including those that are pain-related may hold relevance in pain research and patient care.

### 2.3. Chronic Pain and Biomarkers of Individual Health Status

Over the past decade, biomarkers of individual health status have been investigated in different populations and clinical studies [32,33,34,35,37,38,48,98,99,100,101,102,103,104,105,106,107,108,109]. Higher chronic pain severity has consistently been associated with biomarkers of individual health status: a higher clinical multisystem index, thinner brain cortex, and shorter telomere length, all indications of higher allostatic load [33,102,103,104,105,110]. However, there is more to the story. A study of adults with or at risk for knee osteoarthritis found that although similar levels of chronic pain severity were reported by study participants, with non-linear modeling applied, clinically and statistically significant differences were observed in bilateral temporal lobe brain thickness [102]. The findings highlighted that factors beyond chronic pain severity shape the biological differences observed between people living with chronic pain.

A follow-up investigation was completed to determine the relationships between socioenvironmental factors in the observed bilateral temporal lobe brain structure differences. Socioenvironmental factors play a significant role in an individual’s life experience and are well recognized for their contributions to heterogeneity in pain-related outcomes. Drawing on an approach from a Centers for Disease Control and Prevention study [111], an additive measure of evidence-based socioenvironmental risk factors (education, poverty level, marital status, insurance, employment, and area deprivation index) was investigated in combination with chronic pain severity [34]. Four groups were identified: low socioenvironmental risk/low pain severity, low socioenvironmental risk/high pain severity, high socioenvironmental risk/low pain severity, and high socioenvironmental risk/high pain severity. The combination of high socioenvironmental risk and high chronic pain severity was associated with thinner bilateral temporal lobes compared to the other three groups [34]. In the same sample, the clinical multisystem index was investigated, and the pattern of findings mirrored those observed in brain structure across the four groups [35]. The group with the thinnest bilateral temporal lobes also had the relatively higher clinical multisystem index. The biomarker findings across the two studies showed one group of individuals had a lower biological health status, higher allostatic load, and a greater vulnerability to poor health outcomes compared to their peers [34,35].

Similar to socioeconomic factors, behavioral and psychosocial factors are associated with pain and physical functioning in adults with chronic pain and explain variability in biomarkers of individual health status. Two different studies investigated relationships between a combined profile of chronic pain severity and a psychosocial risk factor with telomere length [104,107]. One study investigated low and high chronic pain severity with low and high perceived stress in individuals reporting knee pain [104]. Individuals reporting high pain and high perceived stress had significantly shorter telomeres compared to the individuals in the other three groups (low pain/low stress, low pain/high stress, and high pain/low stress). The second study investigated low and high pain severity with low and high symptoms of depression in individuals with fibromyalgia [107]. Similar to the first study, the second study also showed those individuals who reported high pain severity and high depression had significantly shorter telomeres compared to the other three pain severity/depression groups [107].

Consistent and complementary findings have also emerged from studies using empirically supported, clinically validated, additive measures of behavioral and psychosocial protective factors (e.g., optimism, social support, tobacco free, healthy BMI, adequate sleep). Across studies, each additional protective factor was associated with better biological health, indicating lower allostatic load. This additive pattern was observed in three biomarkers of individual health status: telomere length, pain-related brain structure, and predicted structural brain age [32,37,38].

## 3. Discussion

### 3.1. Relevance of Findings

Supplemental Table S1 summarizes supporting evidence from our work and that of others. Pain conditions represented in the table span pelvic pain, migraines, chronic low back pain, temporomandibular disorder, fibromyalgia, knee osteoarthritis, and general chronic pain. In addition to the two population studies, investigations from our lab include two multisite clinical studies, UPLOAD and UPLOAD2 (description in Supplemental Table S1). Both studies focused on individuals with primary knee pain with most participants reporting multisite pain. The data across all the references cited indicate that biomarkers of individual health status align not only between different chronic pain conditions but also within two study samples across different biomarkers of individual health status. The coherence between studies and within studies across biomarkers of individual health status is noteworthy and uncommon among the plethora of biomarker research in chronic pain [7,40,41,42]. The pattern of findings aligns with the allostatic load model and suggests promising implications for pain research as biomarkers of individual health status appear to provide a “biological signature of the whole person living with chronic pain.”

Although yet to be determined, biomarkers of individual health status may convey valuable information to help explain treatment response variability and missing considerations in current clinical trial investigations [112,113,114,115]. For example, a numeric rating scale (NRS) of 4 or greater on a 0–10 scale is currently the recommended inclusion criterion for chronic pain clinical trials [116,117]. A recent pain rating does not convey an individual’s biological health status. Hypothetically and consistent with experiences across medical care, a person with a poor biological health status would not be expected to have a similar response to the same dose and duration of a treatment intervention as an individual with an adaptive biological health status [118,119]. Additionally, biomarkers of individual health status might serve as monitoring and surrogate clinical endpoints. Biological measures that are responsive to changes in life exposures and experiences may help meet pressing clinical and research limitations by providing an “inside view” of responses to differing clinical interventions.

### 3.2. A Role for Biomarkers of Individual Health Status

Biomarkers of individual health status are not specific to chronic pain. Rather, they can provide information that may help explain individual differences among people living with chronic pain conditions. A potential role for biomarkers of individual health status could be to complement mechanistic pain biomarkers, providing a more comprehensive understanding of an individual’s pain condition and whole person health status.

The BEST biomarker working group identified the multicomponent biomarker as one of eight different types of biomarkers [4]. Multicomponent biomarkers are described as functioning in two different capacities: integrative and multiplex. An integrative multicomponent biomarker is a biomarker resulting from a combination of measures that inform a single interpretive value. One of the biomarkers of individual health status, the multisystem index, is an example of an integrative multicomponent biomarker. A multiplex multicomponent biomarker comprises independent biomarkers that when combined provide an interpretative classification. Biomarkers of individual health status in combination with pain specific biomarkers could serve as multiplex multicomponent biomarkers. A simplified example follows. Once the type of chronic pain condition is identified, i.e., nociceptive, neuropathic, or nociplastic; biomarkers of individual health status could add to the pain condition classification by providing additional information indicating an individual’s biological health reserve (i.e., low, moderate, or high allostatic load). The identification of an individual’s health reserve would help explain within chronic pain condition variability, identify those more vulnerable to poor outcomes, and assist with determining personalized treatment needs to optimize patient care. With emerging technologies and the broad array of pain biomarker investigations underway, a variety of investigative opportunities for combining measures in the development of multiplex multicomponent biomarkers exist.

## 4. Additional Considerations, Limitations, and Future Directions

A few important considerations are warranted in advance of further investigations on biomarkers of individual health status in chronic pain. First, although measures of chronic pain and phenotyping efforts have improved, existing measures and classifications remain poor predictors of biological measures [7,40,41,42,120]. Findings show that the distinction between low-impact chronic pain and high-impact chronic pain differentiates individuals with more functionally debilitating chronic pain from those with less debilitating chronic pain [121]. However, the classification does not account for factors driving biological changes, including persistence/frequency of symptoms, intensity of symptoms, duration of symptoms or how widespread the symptoms. For example, two people could meet high impact chronic pain criteria, one person has had intermittent, moderate intensity chronic pain in one body site for 12 months, and the other person has had persistent, high intensity multisite chronic pain for many years. The neurobiological and physiological measures of these individuals would differ [33,100,102,105,110]. The relevance of pain domains is further supported by post-surgical investigations showing when pain symptom frequency, intensity, and duration are reduced or eliminated, neurobiological changes are observed [96,97]. Pain measures with a combined consideration for pain frequency, intensity, duration, and number of pain sites will improve interpretation of biomarker findings [105].

Second, a more comprehensive conceptualization of allostatic load will improve communication and understanding. Often represented solely as a stress system measure, allostatic load is a theoretical construct that explains the dynamic biological and biographical interaction during an individual’s life course resulting in their unique health status profile [28]. Although allostatic load develops in response to persisting, unremitting stress without adequate recovery, the array of factors contributing to allostatic load extends beyond the stress response to include all factors relevant to a person’s life experience consistent with the concepts underlying whole person health [28,39].

Similarly, implementation of biomarkers of individual health status into research and clinical care requires some additional considerations. A broader view is needed. Biomarkers of individual health status are not specific to chronic pain; the measures convey the whole person living with chronic pain. Importantly, a plethora of studies on chronic pain have investigated biomarkers of individual health status, e.g., brain structure, brain function, structural brain age gap, and telomere length, and have focused on pain specific relevance covarying for other contributing factors. As biomarkers of individual health status are influenced by the “whole-person milieu,” considerations for a broader view and conceptual shift might offer new information and help address some persisting gaps in pain research and patient care.

Third, the multisystem index, one of the biomarkers of individual health status, comprises representative measures from multiple regulatory systems, e.g., cardiovascular, immune, metabolic, and neuroendocrine. Based on the number of measures outside clinical or relative normal ranges, a total index is determined. The index conveys overall multisystem functioning, biological health status, and related level of allostatic load, Figure 3 [87,88,89]. The relevance of the multisystem index has been scientifically debated and frequently discounted. The concerns often focus on the selection of measures in the represented systems as not being disease or exposure relevant. The multisystem index is not specific to a particular condition or event. Rather, the multisystem index is a metric of physiological function across multiple regulatory systems. It reflects the current biological health status resulting from the cumulative contributions from the intrinsic and extrinsic factors of an individual’s life experience. Thus, the specific measures in the index are less relevant than inclusion of a combined representation of measures across systems [122].

Fourth, as noted in the studies identified in Supplemental Table S1 and illustrated in Figure 3, there are inverse associations and non-linear, hormesis patterns across many biological measures [123,124,125,126]. Some biomarkers will show greater values with high chronic pain severity and high risk life experiences (higher clinical lab-derived multisystem index and older predicted structural brain age) while others will show low values indicating decline in functioning (lower brain activation, thinner gray matter, shorter telomere length). A missed appreciation for these relationships could result in misinterpretation of findings. Research investigations are often based on linear statistical analyses, resulting in the limited ability to observe the inverted U or J curve distribution and missing the opportunity to identify the individuals with the more compromised health status [120]. Moving forward, inclusion of statistical approaches that allow for consideration of non-linear relationships will be helpful.

Fifth, overcoming practical barriers and adding steps to promote rigor and reproducibility will be necessary. The current understanding of allostatic load and biomarkers of individual health status have developed over the past 30 years [69,70]. We draw on a few key findings that can help guide inclusion in pain biomarker efforts. When implementing the multisystem index, a recent publication has identified components that could serve in a consistent multisystem index panel [122]. Next, determining the index score based on validated clinical lab values compared to sample specific quartile splits will be necessary for clinical practice and is an encouraged approach for research investigations. Additionally, studies are underway to optimize commonly drawn lab measures with the goal of identifying whether versions of the multisystem index can be completed that will reduce costs and patient burden. Further, for pre/post measure interpretation, assay processing by the same clinical lab and the use of standardized clinical values will yield the strongest measures. Finally, when possible, the inclusion of two different biomarkers of individual health status (including across differing biological levels of analysis) in investigations will help improve the interpretability of findings and determination of an individual’s biological health status and related allostatic load [29].

Finally, machine learning and artificial intelligence approaches provide exciting opportunities to advance pain biomarker research. Recent investigations and recommendations suggest combining pain-related predictors (socioenvironmental, psychosocial, behavioral, and genetic) with associated biological outcomes in the development of pain-related “composite signatures” [6,7,8,15]. Established biological principles and evidence-based theoretical constructs convey the relationships depicted in Figure 3 [55,127]. With efforts in early stages of investigation, it will be beneficial to evaluate different modeling approaches to determine the most informative and strongest designs [128].

In summary, despite considerable progress in chronic pain research, deficits remain in the identification of pain biomarkers to address research and clinical care needs. Biomarkers that can capture the biological signature of the whole person living with chronic pain hold promise to complement current efforts. Expanding pain biomarker science to include biomarkers of individual health status has the potential to help explain clinical heterogeneity, identify those individuals more vulnerable to poor outcomes, and potentially predict treatment response variability and assist with clinical monitoring and surrogate endpoint needs.

## Data Availability

No new data were created or analyzed in this study. Data sharing is not applicable to this article.

## Supplementary Materials

The following supporting information can be downloaded at: https://www.mdpi.com/article/10.3390/biomedicines14102217/s1, Table S1: References Cited—Chronic Pain and Biomarkers of Individual Health Status.
