# Supplementary material for: Biomarkers of Individual Health Status Provide a Biological Signature of the Whole Person Living with Chronic Pain
Source: Biomedicines. 2026 Sep 30;14(10):2217. doi: 10.3390/biomedicines14102217 (PMC13701456; doi:10.3390/biomedicines14102217)
Supplement: Supplementary file 1 [file biomedicines-14-02217-s001.zip › biomedicines-4432000-supplementary.pdf]

Supplemental Table S1. References Cited - Chronic Pain and Biomarkers of Individual Health Status

Brain Structure, Function, & Structural Brain Age Gap						
**Title**	**Author(s)**	**Year**	**Study Type**	**Population & Pain Condition**	**Biomarker(s) of Individual Health Status**	**Findings**
Increased gray matter density in young women with chronic vulvar pain	Schweinhardt et al	2008	Cross-sectional	n=28 women<br>n=14 with **chronic vulvar pain** and<br>n= 14 **healthy controls**	Total gray matter volume and regional gray matter density	Young women with chronic vulvar pain had significantly higher gray matter densities in pain-modulatory and stress-related areas. Contrasted to prior findings in older women with chronic pain showing lower gray matter densities.
Abnormal gray matter aging in chronic pain patients	Moayedi et al	2012	Cross-sectional	n=34 adults<br>n=17 patients with **non-traumatic temporomandibular disorder – TMD** and<br>n=17 **pain free/healthy controls**	Grey matter volume, cortical thickness, and relationship between grey matter and aging	TMD patients had accelerated whole-brain grey matter atrophy, compared to controls.
Common hippocampal structural and functional changes in migraine	Maleki et al	2012	Cross-sectional	n=30 matched adults<br>n=10 **high frequency migraines - HF**<br>n=10 **low frequency migraines - LF**<br>n=10 **healthy controls - HC**	Hippocampal morphometric and functional differences in response to heat stimuli	Low frequency migraine group had greater bilateral hippocampal volume compared to the other two groups HF and HC. Greater deactivation and lower functional connectivity were indicated in the High frequency migraine group.
Cortical dynamics of movement-evoked pain in chronic low back pain	Wang et al	2020	Cross-sectional	n=55<br>n=27 with **chronic low back pain**<br>n=28 **pain free, age matched controls**	Neural activation during movement-evoked pain	Individuals with chronic low back pain had longer reaction times, delayed peak velocity, greater movement variability, and attenuated reduction in beta power in the premotor cortex and supplementary motor areas compared to controls.

Relationships Between Chronic Pain Stage, Cognition, Temporal Lobe Cortex, and Sociodemographic Variables	Tanner et al	2021	Cross-sectional<br><br>*UPLOAD2 study*	n=147 adults **with or at risk for knee osteoarthritis**<br><br>**Total pain sites**<br>mdn = 5	Cortical thickness in the dorsolateral prefrontal cortex; average cortical thickness in the entorhinal cortex, fusiform gyrus, inferior and middle temporal gyri	Individuals who had higher chronic pain stage and greater sociodemographic risk factors exhibited thinner temporal lobe.
Resilience, pain, and the brain: Relationships differ by sociodemographics	Tanner et al	2021	Cross-sectional<br><br>*UPLOAD2 study*	n=166 adults reporting **chronic pain at ≥1 body site, majority with knee pain**<br><br>**Total pain sites**<br>m=5	Cortical thickness in postcentral gyrus, insula, medial orbitofrontal, anterior cingulate gyrus, and rostral middle frontal gyrus; amygdala and thalamus volumes	Greater number of protective behavioral and psychosocial factors were related to lower reported clinical pain and better pain-related functioning, Patterns differed by sociodemographic groups with structural findings in the right amygdala, bilateral thalamus, and postcentral gyrus.
Chronic Pain Severity and Sociodemographics: An Evaluation of the Neurobiological Interface	Tanner et al	2022	Cross-sectional<br><br>*UPLOAD2 study*	n = 142 adults **with/at risk for knee osteoarthritis**<br><br>**Total pain sites**<br>m=6	Cortical thickness in dorsolateral prefrontal cortex, insula, somatosensory cortex; subcortical volumes (amygdala, hippocampus, thalamus)	Greater chronic pain severity was associated with thinner cortical regions. Sociodemographic differences were indicated in pain-brain relationships.
Greater socioenvironmental risk factors and higher chronic pain stage are associated with thinner bilateral temporal lobes	Antoine et al	2023	Cross-sectional<br><br>*UPLOAD2 study*	n=147 adults **with or at risk for knee osteoarthritis**<br><br>**Total pain sites**<br>mdn=5	Cortical thickness in dorsolateral prefrontal cortex; average cortical thickness in entorhinal cortex, fusiform gyrus, inferior and middle temporal gyri	Combining socioenvironmental risk and chronic pain stage: higher socioenvironmental risk and higher chronic pain stage was associated with thinner bilateral temporal lobe cortex, demonstrating an additive (cumulative load) effect.

Environmental and sociocultural factors are associated with pain-related brain structure among diverse individuals with chronic musculoskeletal pain: intersectional considerations	Domenico et al	2024	Cross-sectional<br><br>*UPLOAD2 Study*	n=147 adults **with knee pain**<br><br>**Total pain sites**<br>mdn = 6	Pain-related brain regions	Sociocultural and environmental factors, e.g., income, social support, experiences of discrimination, and insurance status, help explain variance in pain-related brain structures
More than chronic pain: Behavioural and psychosocial protective factors predict lower brain age in adults with/at risk of knee osteoarthritis over two years	Tanner et al	2025	Cross-sectional and longitudinal over 2 years<br><br>*UPLOAD2 study*	n = 197 adults **with/at risk for knee osteoarthritis**<br><br>**Total pain sites**<br>mdn = 4<br><br>2 year longitudinal subset n = 128	Structural brain age and brain age gap (BAG; predicted − chronological age)	Higher chronic pain stage and greater socioenvironmental risk were associated with older predicted brain age.<br>Greater behavioral/psychosocial protective factors related to younger predicted brain age.<br>Longitudinally, greater number of baseline protective factors predicted lower brain age over 2 years, showing a buffering effect against brain aging.
Multisystem Index						
**Title**	**Author(s)**	**Year**	**Study Type**	**Population**	**Biomarker(s) of Individual Health Status**	**Findings**
Investigating the Burden of Chronic Pain: An Inflammatory and Metabolic Composite	Sibille et al	2016	Cross-sectional	n=12,981 adults in Tromsø Norway<br>**Tromsø Study**<br><br>n=4,545 (35%) with **multi-site chronic pain**	Multisystem index comprised of body mass index, fibrinogen, C-reactive protein, and triglycerides	In a dose response fashion, higher chronic pain stage was associated with a higher multisystem index, suggesting greater biological burden, aka allostatic load. Findings remained after controlling for metabolic and inflammatory conditions.

Allostatic load and pain severity in older adults: Results from the English Longitudinal Study of Ageing	Sibille et al	2017	Cross-sectional	n=5,341 adults<br>≥ 50 years<br>**ELSA –**<br>**English Longitudinal Study of Aging**<br><br>**Chronic pain criteria:**<br>*Frequency and intensity*<br>-No/infrequent<br>-mild<br>-moderate<br>-severe	Multisystem index comprised of immune, metabolic, cardiovascular, and neuroendocrine measures	Greater pain severity (average intensity of frequent pain) was associated with higher multisystem index indicating greater allostatic load.
Telomere Length						
**Title**	**Author(s)**	**Year**	**Study Type**	**Population**	**Biomarker(s) of Individual Health Status**	**Findings**
Chronic pain, perceived stress, and cellular aging: an exploratory study	Sibille et al	2012	Cross-sectional<br><br>*UPLOAD study*	n=36 older adults<br>n=18 **without chronic knee pain**<br>n=18 **with chronic knee pain**	Telomere length	Individuals with chronic pain and high perceived stress had significantly shorter telomeres compared to those with no chronic pain and low stress.
Accelerated aging in adults with knee osteoarthritis pain: consideration for frequency, intensity, time, and total pain sites	Sibille et al	2017	Cross-sectional<br><br>*UPLOAD Study*	n=136 adults **with and without knee pain**<br><br>**Total pain sites**<br>m=4	Telomere length	Adults with a chronic pain stage of 1 had longer telomeres than those with a chronic pain stage of 5. A dose response pattern was indicated between chronic pain stage and telomere length.
Resilience factors may buffer cellular aging in individuals with and without chronic knee pain	Johnson et al	2019	Cross-sectional<br><br>*UPLOAD study*	n=79 adults **with and without knee pain**	Telomere Length	Evidence-based behavioral and psychosocial factors<br>identified as risk or protective based on clinical norms.<br>Protective behavioral and psychosocial factors were associated with longer telomeres.

Chronic Pain and Premature Aging - The Moderating Role of Physical Exercise	Lahav et al	2021	Cross-sectional	n=116 adult men, majority with a physical disability<br><br>n=67 **chronic pain**<br>n=49 **w/o chronic pain**	Telomere Length	Individuals with chronic pain had significantly shorter telomere length, regular physical exercise attenuated the association.
Findings involving two Biomarkers of Individual Health Status						
**Title**	**Author(s)**	**Year**	**Study Type**	**Population**	**Biomarker(s) of Individual Health Status**	**Findings**
Pain is associated with short leukocyte telomere length in women with fibromyalgia	Hassett et al	2012	Cross-sectional	n=88 women<br><br>n=66 with **fibromyalgia FM**<br><br>n=22 **healthy controls HC**	Telomere length and Brain structure	In those with FM, shorter telomere length was associated with higher clinical and experimental pain and lower gray matter volume. Combined relationship between pain and depression indicated.
Elucidating individual differences in chronic pain and whole person health with allostatic load biomarkers	Mickle et al	2023	Cross-sectional<br><br>*UPLOAD2 study*	n=175 adults **with or at risk for knee osteoarthritis**<br><br>**Total pain sites** mdn = 5	Multisystem index and Pain-related brain structures	Higher multisystem index was associated with greater knee osteoarthritis pathology, lower physical function, and thinner insula cortices with trends for thinner inferior temporal lobes and dorsolateral prefrontal cortices.
Disentangling factors contributing to individual differences and health disparities in chronic pain and whole person health with measures of allostatic load	Mickle et al	2024	Cross-sectional<br><br>*UPLOAD2 study*	n=119 adults **with or at risk for knee osteoarthritis**<br><br>**Total pain sites** mdn = 5	Multisystem index and Bilateral temporal lobe thickness	The pattern of findings in the bilateral temporal lobes (Antoinne 2023) was replicated with higher socioenvironmental risk and higher chronic pain stage showing a trend toward greater multisystem index.

Applying evidence-based cross-disciplinary concepts helps to explain the heterogeneity in pain, function, and biological measures in individuals with knee pain with/at risk of osteoarthritis	Mickle et al	2024	Cross-sectional *UPLOAD2 study*	n=173 adults **with knee pain**<br><br>**Total pain sites**<br>m = 6	Multisystem index and Structural brain age	A combined consideration of predisposing dispositional traits, and an environmental stressor, federal poverty level show Individuals with proactive/protective traits and low environmental stress had the lowest pain, disability, and a positive health status (low allostatic load) and those with sensitive/vulnerable traits and living below the federal poverty level had the highest pain, disability, and showed the poorest health status, indicating high allostatic load.

UPLOAD Study - The Understanding of Pain and Limitations in Osteoarthritis Disease study (IRB201500906) was a cross-sectional study of adults 45 to 85 years old who self-reported as non-Hispanic white (NHW) or non-Hispanic black (NHB) with and without knee pain. The study was conducted at the University of Florida and the University of Alabama at Birmingham between 2010-2017.

UPLOAD2 Study - The Understanding of Pain and Limitations in Osteoarthritis Disease 2 study (IRB201400209) was a four year prospective observational study that included adults 45 to 85 years old who self-reported as non-Hispanic white (NHW) or non-Hispanic black (NHB) with and without knee pain. The study was conducted at the University of Florida and the University of Alabama at Birmingham between 2015-2022.

Total pain sites – Pain more days than not over the past three months was assessed on right and left body regions including hands, arms, shoulders, neck, head/face, chest, stomach, upper back, lower back, knees, legs (other than knees), and/or feet/ankles (0-24 sites).
